# Provincial Dietary Intake Study (PDIS): Prevalence and Sociodemographic Determinants of the Double Burden of Malnutrition in A Representative Sample of 1 to Under 10-Year-Old Children from Two Urbanized and Economically Active Provinces in South Africa

**DOI:** 10.3390/ijerph16183334

**Published:** 2019-09-10

**Authors:** Marjanne Senekal, Johanna H Nel, Sonia Malczyk, Linda Drummond, Janetta Harbron, Nelia P Steyn

**Affiliations:** 1 Division Human Nutrition, University of Cape Town, UCT Medical Campus, Anzio Road, Anatomy, Building, Observatory 7925, Cape Town, South Africa; 2 Department of Logistics, Stellenbosch University, Stellenbosch, 7600, South Africa

**Keywords:** malnutrition, double burden, children 1 to under 10 years old, stunting, wasting, overweight, obesity

## Abstract

The objective of this study was to determine the prevalence and socio-demographic predictors of malnutrition in two urbanized economically active provinces (Gauteng N = 733, Western Cape N = 593) in South Africa. A multistage stratified cluster random sampling design was applied. Fieldworkers visited homes, measured children aged 1-<10-years old (N = 1326) and administered a questionnaire (mother/primary caregiver). In under-five year old children (N = 674) 21.6% were stunted [height-for-age z-score < −2 SD], 5.6 % underweight [weight-for-age z-score < −2 SD], 10.3% overweight (body mass index-for-age z-score) (BAZ)> +2 SD ≤ +3 SD] and 7.0% obese (BAZ > +3 SD). In 5–<10-year olds (N = 626) 6.7% were stunted, 6.8% underweight, 13.4% overweight and 6.8% obese. Stunting and overweight in the same child was present in 5.7% under-five year olds and 1.7% in 5–<10-year olds. Multiple logistic regression analyses identified having a mother with a post-grade 12 qualification (OR = 0.34) and having an obese mother (OR 0.46) as protectors and being in the under-five age group (OR = 3.73) as a risk factor for stunting. Being in the under-five age group was also a risk factor for a BAZ > 1 (OR 2.39), while being in the third wealth quintile was protective (OR = 0.62). Results indicate that stunting and overweight/obesity are still present at concerning levels, especially in the under-five age group.

## 1. Introduction

Many countries in sub-Saharan Africa, including South Africa, have both a high prevalence of stunting in children and of overweight and obesity in children and adults, also referred to as the double burden of malnutrition [1]. Stunted children may become obese adults, who in turn appear to be more at risk of developing non-communicable diseases (NCDs) in adulthood [2]. Furthermore, undernutrition and overnutrition have been found in the same household [3]; a study in Benin, for example, showed that child protein energy malnutrition coexisted with maternal overweight/obesity in 16.2% of 148 households in poor neighborhoods. The authors theorized that child malnutrition and maternal overweight could both stem from poor socioeconomic conditions, lack of sanitation, and poor dietary variety [3]. According to de Onis and Branca [2], childhood stunting is the best overall indicator of children’s well-being and an accurate reflection of social inequalities.

The number of overweight or obese children in the under-five age group has increased from 32 million globally in 1990 to 41 million in 2016 [4]. According to the World Health Organization (WHO) [4], the majority of overweight or obese children live in developing countries and the rate of increase has been more than 30% higher than that of developed countries. In the WHO African Region, the number of overweight or obese children has increased from 4 to 9 million from 1990 to 2016.

A study by Wijga et al. on a large cohort of Dutch children showed that childhood obesity is associated with numerous childhood health problems including a lower general health score, more school absenteeism, and more visits to a general practitioner [5]. Obesity was also significantly associated with bronchitis and the use of antibiotics. Childhood obesity is also associated with numerous age-related consequences; these include an increased risk of developing cardiovascular disease and metabolic syndrome, type 2 diabetes, obstructive sleep apnea, polycystic ovarian syndrome, asthma, infertility, non-alcoholic fatty liver disease, orthopedic complications, increased rates of cancer, psychiatric disease, among others [6].

The double burden of malnutrition in South Africa is illustrated by the finding that the prevalence of stunting (height-for-age z score (HAZ) < −2 SD) in South African children under-five years old was 27.4% in 2016 [7]. Furthermore, the prevalence of overweight and obesity (weight-for-height z score (WHZ) > +2 SD) in this age group was found to be 13.3% in the same year [7] and 7.2% in boys and 16.4% in girls in 2012 [8] in children 5−9-years. Recently, researchers have pointed out that some studies show that stunting and overweight are not necessarily mutually exclusive and refer to the co-existence of these forms of undernutrition in the same child as phenomenon as stunted and overweight [9]. They propose that this phenomenon may reflect a new layer of malnutrition that is linked to rapid nutrition transitions occurring in low to middle income countries.

A mother’s preconception weight and weight gain in pregnancy are two very important determinants of childhood obesity [10]. Maternal obesity and excessive gestational weight gain are associated with fetal macrosomia and childhood obesity, and this effect extends into adulthood [11]. When taking into consideration the finding that 62% of women in the 15 to 49-year group in South Africa are overweight or obese [7], as well as evidence that shows that overweight and obese women have the highest prevalence of excessive weight gain during pregnancy [12], it follows that there is a large risk of childhood obesity in their offspring.

National South Africa surveys that reported on malnutrition in children under 10-years old include the 1994 South African Vitamin A Consultative Group survey in children 6–71 months [13], South African Demographic and Health Survey (SADHS) of 1998 [14]; the National Food Consumption Survey (NFCS) in 1999 (1–9-year olds) [15], the SADHS of 2003 (under-five year olds) [16], the National Food Consumption Survey-Fortification Baseline (NFCS-FB-1) in 2005 (1–9-year olds) [17], the South African National Health and Nutrition Survey (SANHANES) in 2012 (2–14-year olds) [8] and the 2016 SADHS (under-five year olds) [7]. In order to contribute to further insights into trends in the profile of malnutrition over time in the vulnerable under-five year age group, but also children in the 5–<10-year old age range, the aim of the current study was to look at the prevalence of the dual burden of malnutrition in a representative sample of 1–<10-year old children in two urbanized and economically active provinces in South Africa. We also investigated sociodemographic predictors of stunting and overweight/obesity in these children.

## 2. Materials and Methods 

### 2.1. Study Area

The two provinces selected were Gauteng (GTG) and the Western Cape (WC), because they are the most rapidly urbanizing and wealthiest provinces, with extensive migration from rural areas to cities in search of jobs and a better quality of life [18].

### 2.2. Structure of the Sample and the Sampling Procedure 

The sampling strategy of the current provincial dietary intake study (PDIS) incorporates a multistage stratified cluster random sampling design, using the methodology applied in demographic and health surveys as described in the Sampling and Household Listing Manual, USAID 2012 [19]. The PDIS in the Western Cape included 17 urban formal enumerator areas (EAs), 10 urban informal EAs, and 10 rural EAs. Gauteng had 26 urban formal EAs, 10 urban informal and 10 rural EAs.

The formula included below was used to determine the number of households per stratum, for six domains: two provinces each with urban formal, urban informal and rural areas:

N = Deft^2^ × { [(1/P) − 1]/a^2^ }/(R_1_ × R_2_ × d) (1)

where N is the final sample size in terms of the number of households per stratum while taking non-response into account: N (= 175) is the sample size of households; Deft (= 1.3) is the design effect; P (= 0.21) is the estimated proportion of children classified as stunted; a (= 0.2) is the desired relative standard error; R_1_ (= 0.96) is the individual response rate; R_2_ (= 0.89) is the household gross response rate; and d (= 1.06) is the number of eligible individuals per households. It was proposed to survey 175 × 6 strata, or 1050 households.

For the precision of estimates to be acceptable across regions including different age groups, experience shows that a minimum of 50 interviews per group are needed so that reliable estimations for indicators under investigation can be obtained. The final sample allocation reflects a power allocation of 0.5, which is between the proportional allocation and the equal size allocation, so that the survey precision in the urban formal areas is comparable with the urban informal and rural areas, with urban informal and rural areas slightly over-sampled. Since the sample sizes of Gauteng rural, Western Cape rural and urban informal were less than 150, we increased sampling accordingly to ensure sufficient observations per cell in each age group, with the proposed sample size then being 1050 + 218 = 1268.

Sampling weights were calculated to adjust for the oversampling in the rural and urban informal areas and the number of children in the 1–<5 and 5–<10-year age groups, bearing in mind the survey design. The final weight was the product of the proportional and realization weights. The final post-hoc stratification weighting reflects the census population of the Western Cape and Gauteng provinces.

### 2.3. Selection of Households

Maps of relevant primary sampling units (PSUs) were generated and passed on to the respective fieldwork teams. An estimate was made of the total number of households (HHs) in each EA to determine the approximate number of qualifying HHs with children within the prescribed age interval in the EA. A listing of eligible households was compiled in all selected EAs, which served as a sampling frame for the selection of households. HHs (a maximum of 16) were then selected based on a predetermined fixed interval (calculated to be specific to each EA) starting from a randomly determined point. A backup sampling frame was constructed in each EA by asking members of the 16 selected HHs to identify nearby HHs with women and children of the appropriate age.

### 2.4. Selection of Children Within Households

One child in each randomly selected HH was included in the survey. If there was more than one child present in the prescribed age interval within a HH, then all eligible children in the HH in age order were numbered for random selection of one child using a “Random Number Table” designed for this purpose. 

The inclusion criteria for the current study were as follows: children aged 1–<10-years old; male or female; availability of a parent/primary caregiver to provide consent; and availability of a parent/primary caregiver to assist with completion of the research questionnaires. The exclusion criteria were as follows: children who were mentally or physically handicapped; children who were on a prescribed diet e.g., for Type 1 diabetes; children who were ill at the time of the visit or were ill during the past 24 h; children whose mothers/caregivers were unable to respond to, or appeared to be incapable of responding or providing reliable information; children whose mother/caregiver was under the influence of alcohol/drugs or was under-15 years old.

### 2.5. Fieldwork Teams

Each province was led by a provincial dietitian who was responsible for the overall management of the research teams in the two provinces. Two field work teams of five members each were deployed in both GTG and the WC; each team included a team leader and two pairs of field workers. Team leaders and field workers received a week-long extensive theory and practice training session, facilitated by experienced researchers in anthropometric measurements, dietary intake methodology, as well as the administration of sociodemographic questionnaires. The field workers were selected based on a minimum level of grade 12 schooling, as well as other experience in survey type field work.

### 2.6. Measures

#### 2.6.1. Socio-Demographic Questionnaire

The questionnaire comprised questions about the child: birth date, gender, birth order, schooling/day care center, and dietary supplements from clinics. Questions about the family and household included: head of household, primary caregiver, marital status of mother, educational status of mother and father, employment status of mother and father, type of house, availability of electricity or other energy devices, drinking water, type of toilet, and household density. 

A wealth index was calculated as suggested by the World Bank [20] and applied in the 2016 South African Demographic and Health Survey [7]. The wealth index was based on assets and amenities available in the home and environment and was developed using an iterated principal factor analysis. The wealth index was constructed from 21 items (Supplementary Table S1) that had a loading on the first three factors of greater than 0.3 or less than −0.3. The binary items with positive loadings on factor 1 are: have electricity, have an electric or gas stove, cook with electricity, have a working refrigerator, have a working TV, flush toilet in house, live in a brick house, tap water inside the house, tap water in yard, flush toilet in yard, own a car, own a washing machine, own a microwave, own a computer, own a watch, own a vacuum cleaner, own a bicycle and the number of rooms in the house as an ordinal variable. The following items have negative loadings on factor 1: drinking water comes from a communal tap, cook with paraffin and finally, the structure of the house is informal. Households were classified according to the wealth index into quintiles.

#### 2.6.2. Hunger Scale Questionnaire

Hunger (food security) was measured using the Community Childhood Hunger Identification Project (CCHIP) questionnaire [21]. This questionnaire measures household, child and individual level food security using a total of eight questions. If any of the questions are affirmative, a score of one is given. A total score of 5–8 indicates that food shortage is present in the house. A score of 1–4 indicates that the household is at risk of hunger (poor food security) and a score of zero indicates that the house is food secure. 

#### 2.6.3. Anthropometry: Weight of Children and Mothers

Electronic digital scales (Scalerite Micro glass bathroom scales, Scalerite, Benrose, Gauteng, South Africa, capacity 180 kg) were used to determine weight in accordance with standard procedures by trained fieldworkers. These scales were calibrated regularly using standard weights. The children were weighed in light clothing (without coats, cardigans and shoes) and the reading was recorded to the nearest 100 g. The measure was then repeated and the average of the two readings used. The same procedure [22] was followed to weigh the mother.

When children were unable to stand unaided (<24 months), the mother/primary caregiver stepped on the scale and her reading was recorded. The child was then handed to her and she was weighed with the child. The reading of the child’s weight was then recorded by subtracting the mother/caregiver weight from the total weight. Nappies and other heavy clothing were removed before the child was weighed [22].

#### 2.6.4. Anthropometry: Height of Children and Mothers

The height of children older than two years and mothers was measured without shoes using a stadiometer (SECA 213 portable stadiometer, SECA, Hamburg, Germany), which was placed on an even surface. Children and mothers stood on the base board with their backs to the vertical rod of the stadiometer, facing the fieldworker. Children and mothers stood upright with shoulders relaxed and heels against the measuring board. Arms hung loosely by the side and the head was in the Frankfurt plane. The fieldworker then lowered the headboard until it touched the head (any hairclips/pieces that may have impacted the reading were removed prior to taking the measurement). The reading was taken to the nearest 0.1 cm. The measurement was then repeated, and the average used [22]. 

Children who were <24 months old had their supine length measured on a measuring mat (SECA 210 mobile measuring mat for babies and toddlers, SECA, Hamburg, Germany). This involved the fieldworker and the mother/primary caregiver placing the child on his/her back on the board. The crown of the head touched the top of the head board. The mother/primary caregiver then held the child in a flat position with legs straight while the footboard was moved up to touch the heels. Length was measured to the nearest 0.1 cm. The measurement was repeated twice, and the average used [22].

#### 2.6.5. Anthropometry: Mid-Upper Arm Circumference (MUAC) of Children

The mid-upper arm circumference (MUAC) was measured with a non-flexible tape (SECA 201 ergonomic circumference measuring tape, SECA, Hamburg, Germany) at the point on the left arm midway between the lateral border of the acromion and the olecranon when the arm was flexed at 90 degrees. The reading was taken to the nearest 0.1 cm. Two measurements were taken, and the average used [22].

#### 2.6.6. Interpretation of Anthropometric Measures

The WHO growth standards [23] were used to calculate height-for-age z scores (HAZ) (all children), weight-for-age z scores (WAZ) (all children), body mass index z scores (BAZ) and mid-upper arm circumference z scores (MUACZ) (1–<5-years only). Prevalence of stunting, underweight, wasting, overweight and obesity were determined based on the WHO growth standards [23,24]. BAZ was used to determine the prevalence of underweight, at risk of overweight, overweight and obesity in 1–<5-year olds and underweight, overweight and obesity in 5–<10-year olds [24]. Cut-offs applied for these purposes are delineated in Table 1. Body mass index (BMI) was calculated for the mothers as weight divided by height squared (kg/m^2^) and classified as underweight (<18.5), normal weight (18.5–24.9), overweight (25–29.9) or obese (≥30) [25].

### 2.7. Data Analyses

After completion of an EA, the questionnaires were checked by the two provincial dietitians who managed the fieldwork as well as the quality control of data collection in each province. The questionnaires were then dispatched to a central point for data entry. Data analyses were conducted using SAS Version 9.4, SAS for Windows (SAS Institute, Cary, NC, USA). Weighted means, proportions and 95% confidence intervals were calculated by incorporating the strata and cluster structures in the data. The prevalence of stunting, underweight, wasting, overweight and obesity were calculated for children and prevalence for overweight and obesity for the mothers using the mentioned indicators and cut-offs.

The Chi-square test was used to test for relationships between nominal variables such as province, different residential areas and age groups. The independent t-test was used to test for significant differences between the two provinces for variables such as the number of persons in the household and the number of rooms in the house, respectively.

Firth’s bivariate logistic regression was performed to identify socio demographic factors associated with stunting, at risk of overweight, overweight, obesity and the burden of being both stunted & overweight. Variables in the model included: who looks after the child; age and gender of the child; head of the household; marital status of the mother; mother’s employed; father’s employed; mother’s education matric or more; father’s education matric or more; BMI of the mother of the child; wealth index quintiles; province; type of residence; race; and finally the risk of hunger classification.

Significant relationships (*p* < 0.05) were further investigated in multivariate logistic analyses. Odds ratio estimates and profile-likelihood 95% confidence intervals are reported when using the bivariate and multivariate logistic regressions. The Wald chi-square was used to test the significance of the estimates obtained in the logistic regressions. The Hosmer and Lemeshow goodness-of-fit test indicated that all the multivariate models were a good fit.

### 2.8. Ethics

The study was approved by the University of Cape Town Faculty of Health Sciences Human Research Ethics Committee (HREC REF: 326/2018). Parents or primary caregivers of children provided informed, signed consent. In addition, children aged 6–<10-years provided verbal assent. The study was conducted in accordance with the principles of the 2013 Declaration of Helsinki, Good Clinical Practice (GCP) and the laws of South Africa [26].

## 3. Results

In 2018, 733 children were studied in GTG and 593 children in the WC (combined N = 1326) (Table 2). The person who looked after the child most of the time was the mother, followed by a grandparent. The father was most likely to be the head of the household, followed by a grandmother and to a lesser degree the mother or the grandfather. Thirty-nine percent of mothers were unmarried, and 53.4% of mothers and 29.1% of fathers had less than grade 12 as their highest level of education. Only 28% of mothers were employed, while 64.9% of fathers were employed. Of the total sample, 74.4% were Black African and 24.1% were of mixed ancestry. Twenty-five point five percent of mothers were overweight and 42.6% were obese. Altogether, 20.8% of households indicated a food shortage. 

There were significant provincial differences for most of the sociodemographic characteristics with exception of the age and the gender of the participants, as well as the father’s employment status and highest level of education. Compared to mothers from GTG, mothers in WC were significantly less likely to be the head of the household, to have a grade 12 qualification, to be unmarried and to be unemployed, and significantly more likely to be obese.

**Figure 1:**
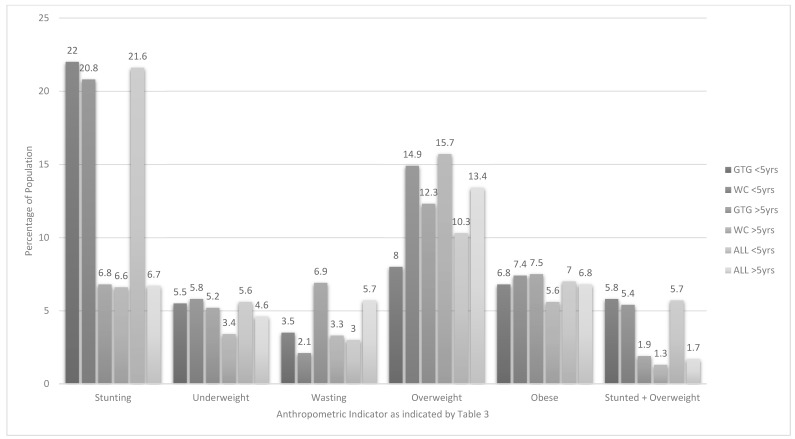
Prevalence of stunting, underweight, overweight, obesity and stunted & overweight in children aged 1–<10-years old in Gauteng (GTG) and Western Cape (WC).

As can be seen from Table 3 in children 1–<5-years old, 21.6% were stunted (HAZ) of whom 6.9% were severely stunted. Interpretation of BAZ shows that 20.4% were at risk of being overweight, 10.3% were overweight and 7% were obese. The combination of stunting & overweight was present in 5.7% of the total group of 1–<5-year olds. Children in this age group from the WC were significantly more likely to be overweight than those from GTG, although the risk for being overweight or obese was similar between the provinces. Figure 1 shows clearly that stunting was highest in 1–<5-year olds in both provinces and in total. Overweight was high in both 1–<5-year olds and in 5–<10-year olds (10.3% and 13.4%), respectively. Overall, the prevalence of being underweight (WAZ) or wasted (BAZ) or undernourished (MUACZ) was less than 10% (Table 3). There were no significant differences between children in this age group for living in urban formal, urban informal or rural areas for any of the anthropometric indicators. However, notable trends in this regard include that the highest prevalence of stunting (34.5%) was found in urban informal areas of GTG, while the highest prevalence of underweight (12.2%) was found in rural areas in both GTG and WC. The highest prevalence of overweight (15.7%) was found in WC urban formal areas and the highest prevalence of obesity (12.7%) in WC urban informal areas (Table 3).

In children 5–<10-years old 6.7% were stunted (HAZ) of whom 2.1% were severely stunted. In terms of overweight and obesity 13.4% of children were overweight (BAZ) and 6.8% were obese. The prevalence of being both stunted & overweight was 6.8%. In line with results for the 1–<5-year olds, the prevalence of underweight (WAZ) and wasting (BAZ) was less than 10% (Table 3). There were no significant differences between children in this age group for province or living in urban formal, urban informal or rural areas for any of the anthropometric indicators. However, in children 5–<10-years old notable trends in this regard include that the highest prevalence of stunting (15.1%) was found in rural areas in the WC. The highest prevalence of overweight (16.5%) was found in the urban informal areas in the WC and of obesity in the urban formal areas in GTG (8.2%) and rural areas in the WC (8.2%).

**Figure 2:**
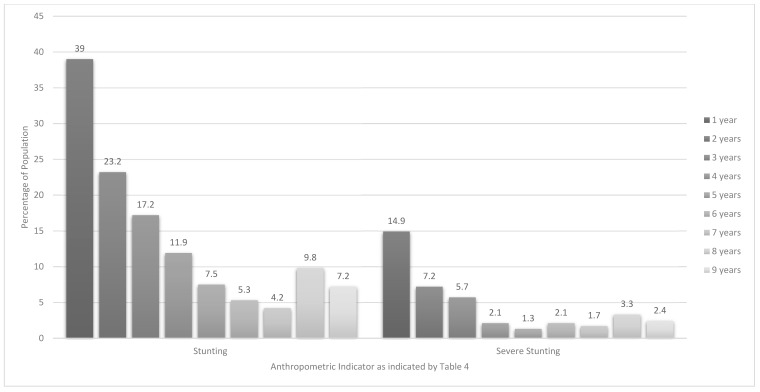
Prevalence of stunting and severe stunting in children aged 1–<10-years by year age groups.

**Figure 3:**
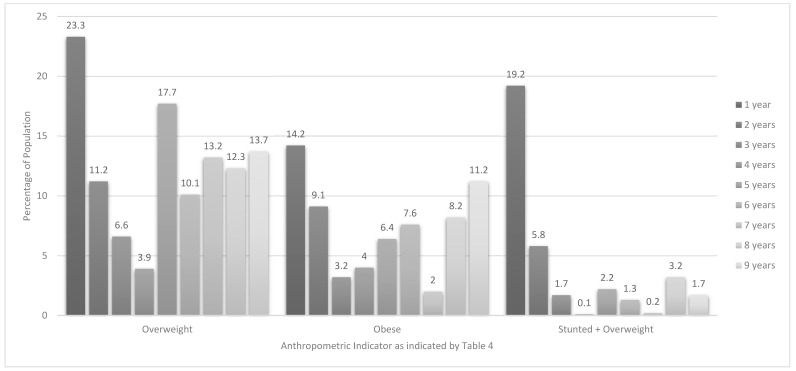
Prevalence of overweight, obesity and stunted & overweight in children aged 1–<10-years by year age groups.

Table 4 and Figure 2 and Figure 3 present anthropometric data on every 12-month age group within the 1–<10-year age range. The highest prevalence of stunting (HAZ) was found in the 1-year old group (39%), followed by the 2-year olds (23.2%), and 3-year olds (17.2%). The highest prevalence of wasting (BAZ) was found in 6-year olds (9.8%). The highest prevalence of overweight and obesity in 1–<5 year olds were found in 1-year olds at 23.3% and 14.2%, respectively. The highest prevalence for overweight in 5–<10 year old children was 17.7% for 5-year olds and for obesity was 11.2% for 9-year olds (Figure 3). The highest prevalence of stunted & overweight was 19.2% in 1-year olds. Significant differences between provinces include a greater likelihood for 3-year olds from GTG to be stunted (9.0% versus 0.2%) and a smaller likelihood for these 3-year olds to be overweight (2.6% versus 13.2%) than their counterparts in the WC (Footnote to Table 4). Regarding gender differences, significantly more 1-year old males (11.0%) than females (0.3%) were stunted and more 8-year old males were severely wasted (7.5% versus 0%). Significantly more 1-year old females (31.4%) were overweight compared with males (13.3%). This was also the case for obesity in 9-year olds (17.3% versus 0.7%). Significantly more 1-year old females (26.4%) than males (10.3%), were stunted & overweight (Footnote to Table 4).

Bivariate logistic regression analysis (Table 5) showed that having a mother with a qualification after matric (OR 0.38, 0.18–0.71), being in the 4th quintile of the wealth index (OR 0.53, 0.30–0.92) and having an obese mother (OR 0.42, 0.28–0.64) were all significantly protective against stunting (HAZ). However, being in the younger age group (1–<5-years)) was highly predictive of stunting (OR 3.77, 2.56–5.68), having a BAZ > +1(OR 2.38, 1.81–3.14) and being both stunted and or overweight or obese (OR 5.97, 3.10–12.78). Being in the 3rd quintile of the wealth index was significantly protective against having a BAZ > +1(OR 0.63, 0.40–0.97) (Table 5). Bivariate logistic regression analysis of items included in the wealth index is presented in Supplementary Table S2. The following factors were shown to be significantly protective against stunting in 1–<10-year old children: having a refrigerator, microwave oven, vacuum cleaner, television, mobile phone, and electricity in the house. Significant predictors of stunting were cooking with paraffin, using a bucket toilet and living in a shack.

When the significant predictors were combined in multivariate regression analysis the 4th quintile wealth index fell away as predictor of stunting, while the 3rd quintile relating to overweight/obesity remained significant (Table 6). The associations between age, having a mother with a qualification after matric and having an obese mother with select anthropometric indicators remained significant.

## 4. Discussion

The results of this survey that was conducted in two urbanized economically active provinces in South Africa confirm that both stunting and overweight/obesity (double burden of malnutrition) remain public health problems of concern, although there is some indication of improvement in stunting and overweight, but not obesity in the two provinces.

With the prevalence of stunting in children in GTG and the WC combined at 21.6%, overweight 10.3% and obesity 7% in the under-five age group, and 6.7%, 13.4% and 6.8% respectively in the 5 –<10-year olds, it is very unlikely that these provinces, and most probably the country as a whole, considering the information provided in Table 7, will be able to meet the United Nation’s Sustainable Development Goal (SDG) 2, target 2.2 of ending all forms of malnutrition by 2030 [27]. The prevalence of underweight (WAZ) of 5.6% and wasting (BAZ) of 3% in under-five year olds and 4.6% and 5.7% respectively in 5–<10-year olds in this study is approaching the 2030 goal of <3%, which is strictly interpreted as levels of malnutrition that could be expected in a healthy population distribution [27]. However, it must be noted that the prevalence of wasting among the eight-year-old boys was significantly higher than in girls (7.5% versus 0%), indicating that more vulnerable subgroups should be identified and specifically targeted for intervention.

Although stunting has been on the decrease in most regions in the world since 2000, Africa still experiences an increase in the number of stunted children [28]. When considering the trends in stunting prevalence in South African children prior to 2018 as depicted in Table 7, bearing in mind the use of different sampling frames, sample sizes and age ranges, a similar situation is evident in the under-five age group in the country. The prevalence of stunting in under-fives increased from 16.1% in 1993 to 33.4% in 2003, after which it decreased, but it seems to have been slowly increasing nationally over subsequent years (26.4% in 2005, 24.9% in 2008, 26.5% in 2012 and 27.4% in 2016) [29,30]. However, the prevalence of stunting found in the current survey points to lower levels of stunting in GTG (22%) and WC (20.8%) in 2018 (no difference between the two provinces or between living areas i.e., urban formal, urban informal and rural) when compared to the 2016 national figures [7]. Stunting prevalence for under-fives in 2016 was 26.5% for GTG and 34.7% for WC [7], indicating that this malnutrition problem has decreased in this age group in these two provinces. If the trend found for the WC continues, namely a reduction of approximately 14% over a two-year period, the WHO world target of 47% reduction in under-five stunting by 2025 may well be achievable in this province. 

Of note is that children in our study younger than two-years (1000 days) were most vulnerable, with 39% of the one-year olds and 23.2% of the two-year olds being stunted, compared to 17.2% of the three-year olds and 11.9% of the four-year olds. National level comparative data for the younger age group comes from a number of studies. The 1993 SAVACG study [13] reported a stunting prevalence of 23.4% for 12 to 23-month olds, reflecting the much lower prevalence of stunting found for under-fives as a whole at the time (Table 7). Casale [30] reported that 44.9% of 6-month to four-year old South African children were stunted in 2008 (Table 7), which is more in line with our findings. In their investigation of malnutrition in children across thirty-six low- and/or middle-income countries (LMICs) between 1990 and 2012, Tzioumis et al. [31] also showed that children younger than two years old were worse off in terms of stunting than those who were two years or older. They also found that stunting continued to increase in children younger than two years in one-third of the included countries after 2000, whereas few increases were seen in the older age category. These findings are in line with the contention that a child’s linear growth potential is largely determined by the time that they turn two years old; thus chronic poor nutrition during the first 1000 days of life may result in stunting and as a result negatively affect future growth and health [28,31,32].

It is evident from Table 7 that less national data is available on stunting prevalence in 5–<10-year olds, which seems to be much lower than for the under-five year olds. National data for 7–9-year olds in the 1999 NFCS [15] shows that stunting prevalence was 11.3%, while it was 12.2% for 5–9-year olds in 2008 [30]. The prevalence that we found in 2018 was almost half the 1999 and 2008 figures, with no difference between the two provinces or across residential areas. Unfortunately, no provincial level stunting prevalence data is available for this age group and it is thus not possible to reflect on how the lower levels of stunting in this age group are aligned with provincial level changes over time.

Chronic energy and micronutrient deficiencies that result in stunting may arise as a result of socio-economic challenges (poor households, low education level of parents, young mothers and mothers who have a low BMI), low birth weight, prolonged breastfeeding (> 12 months), diarrhea episodes, being a male child and living in rural areas [33]. A range of recommendations have been made and policies and interventions were rolled out in South Africa over the past 20 years to address undernutrition problems, including support for subsistence farming and small-scale agricultural programmes to boost food production, a Vitamin A supplementation programme, food fortification programmes targeting key micro-nutrients, breastfeeding promotion, child support grants, the National School Nutrition Programme, a National Nutrition Security Development Programme, a Food Security Policy for South Africa, housing improvement and access to healthcare for all [32,34]. Devereux and Waidler [35] argue that the paradox of limited or absent national level improvement in nutritional child stunting rates during the last two decades, despite the implementation of these interventions and improvements in food security, may be linked to the fact that social grants may not be sufficient to cover even basic food needs of a child and moreover have multiple uses and multiple users. 

It can be argued that the reduction of stunting from 2016 to 2018 in the two provinces, but especially the WC, may be the result of effective implementation of one of more of the mentioned initiatives, however, more research needs to be conducted to confirm this possibility. In this study, bivariate regression analyses reiterate the importance of sociodemographic protectors against stunting. Being older (5–<10-year old age group), having a married mother, having a mother with a post grade 12 qualification, having an obese mother and being in a higher wealth index quintile (quintile 4) were identified as protective factors. Age group, having a mother with a post grade 12 qualification and having an obese mother remained significant following multiple regression analysis. This is similar to results from the bivariate regression analyses conducted on the 1999 NFCS data [15]; protectors against stunting were found to be father being the head of household, mother being married, mother having a higher level of education, as well as a number of indicators covered in the PDIS wealth index that point to higher socio-economic status, namely living in a brick/cement house, having more than three rooms in the house, having a flush toilet in the house, having a television and refrigerator in the house and not using paraffin for cooking [36]. Although results on the hunger scale indicated that 20.8% of the total sample from the two provinces had a food shortage in the household, hunger did not emerge as a significant predictor of stunting in the current study, as was the case in 1999 [36]. This may be due to the fact that the two wealthiest provinces were the only ones tested.

The finding that having an obese mother protects against stunting needs to be considered with caution; it may reflect greater accessibility to food (energy) in these households, but having at least one obese parent is known to increase the risk of overweight/obesity in a child on the one hand [37,38,39] and increase NCD risk in the parent on the other [40]. It is in actual fact a concern that 25.5% of the mothers were overweight and 42.6% were obese (no difference between the two provinces), although not unexpected, bearing in mind the overweight and obesity prevalence reported in national surveys conducted over the past 15 years.

Consideration of our 2018 results on being at risk of overweight (under-fives only), overweight or obese for the two provinces within the context of results from national surveys conducted from 1999 shows that the generally upward trajectory of children having a BAZ > +1 in the country may be approaching a turning point in terms of overweight in the two provinces included in the PDIS, although the decrease in overweight may have been offset by an increase in obesity in the under-five age group (Table 7). 

Prevalence percentages taken from Table 7 for discussion purposes are those based on BMI-for-age, with the exception of the 1999 NFCS for which only WHZ and IOTF cut-point [41] information is available. At risk of overweight increased from 13.4% in 1999 (1–3 year and 4–6-year olds WHZ) [36] to 20.1% in 2003 (under-fives) [29], then to 24.9 in 2012 [8], while the 2018 (PDIS) prevalence is lower at 20.4%. Overweight increased from 7.4 (1–3-year olds) and 5.9% (4–6-year olds) in 1999 (WHZ) [36] to 11.3% (under-fives) in 2003 [29] and to 18.1% in 2008 [30], but then decreased to 12% in 2012 [29], with the 2018 PDIS prevalence being 10.3%. Being overweight was significantly higher in the under-fives in the WC than in GTG, but province did not emerge as a significant predictor of having a BAZ > +1 in bivariate regression analysis. Obesity increased from 7.0% (1–3-year olds) and 1.6% (4–6- year olds) in 1999 (WHZ) [36] to 8.5% (under-fives) in 2003 [29] and then decreased to 4.8% in 2008 [30] and to 4% in 2012 [29], with the 2018 PDIS prevalence in under-fives being 7%. It must be noted that a much lower prevalence for all malnutrition indicators was reported for under five-year olds in the 2005 NFCS-FB [29].

In the older age group overweight increased from 9.5% in 1999 (7–9-year olds, IOTF) [36] to 18.2% in 2008 in 5–9-year olds [30], with the prevalence decreasing to 13.4% in 2018 (PDIS). However, the prevalence of obesity seemed to have remained relatively consistent over the years in this age group (6.5% in 1999 (IOTF) [36], 7.5% in 2008 [30] and 6.4% in 2018). Only 0.7% of nine-year-old boys were obese, compared to 17.3% of the girls in our study. This is in line with findings from previous national South African surveys among children and adults that female gender donates a greater risk for being obese [7,16,42]. Despite some indication that overweight may be decreasing in the two provinces included in this study, it is highly unlikely that the target of reduction of obesity by 10% by 2020 as stated in the 2015–2020 Strategy for the Prevention and Control of Obesity in South Africa can be achieved. This despite the fact that one of the six goals formulated in the strategy focusses specifically on children (Goal 4: Support obesity prevention in early childhood (in-utero to 12-years)) [42].

Similar to findings from previous studies [13,36], the 1 year-olds in the PDIS were most likely to be at risk of overweight category (30.6%) or overweight (23.2%) or obese (14.2%). This finding may explain why being in the older (5–<10-year old) age group was identified as a significant protector against having a BAZ > +1 in both bivariate and multiple regression analyses. Interestingly, the gender difference in prevalence of overweight seems to have already been present in one-year olds, as one-year-old girls were more than twice as likely as their male counterparts to be overweight. Tzioumis et al. [31] found in their investigation of malnutrition across 36 LMICs that overweight rates increased in children younger than two years and those who were two years and older, but the rates increased in more countries for the younger than older age group. Furthermore, they found that the magnitude of increase in overweight prevalence was more extreme in the younger age group. However, importantly, the WHO cautions that classification of young children as overweight or obese needs to be done with great caution as children in this group are still growing. Placing young children on energy restricted diets may contribute to other forms of malnutrition and associated health risks [24]. The apparent spike in the prevalence of overweight from 3.9% in four-year-olds to 17.7% in the five-year-olds in our study is the result of the change in cut-offs for the under-fives and 5–19-year old children (overweight in children <5 years: BAZ > 2 and ≤3; overweight in children 5–19: BAZ > 1 and ≤2) i.e., children who are classified as overweight at 59 months would be classified obese at 61 months while they maintain the same z-score [24].

The 2015–2020 Strategy for the Prevention and Control of Obesity in South Africa [42] mentions that the increase in overweight and obesity in the country may be linked to poverty, increased intake of energy dense cheap foods and reduced physical activity levels. It is also mentioned in the document that there may be a significant association between obesity in South Africans and years of completed education, as well as the number of assets owned by the household [42]. However, with the exception of the age of the child, none of the socio-demographic variables investigated in this study, nor residential area (urban formal, urban informal and rural), were found to be predictive of having a BMI > +1, unlike the results reported for predictors of obesity in the 1999 NFCS [36]. The latter study showed that a lower socio-economic status (living in a shack, having a communal source of water, using paraffin for cooking, having no refrigerator or stove in the house and having a mother with a primary school education only) was associated with a reduced risk of obesity in 1–9 year old children. Our finding that there is no difference in overweight/obesity prevalence between rural and urban children does not support the contention that over-fatness is typically a greater problem in urban than rural areas in all South African provinces. This could be attributable to the penetration of the obesogenic food environment into the rural areas of these two provinces, with the traditional high fiber diet being replaced by one that is energy-dense and high in fat, saturated fat, added sugar and salt, and as a consequence less nutrient dense. 

Finally, the co-existence of being stunted and overweight/obese (stunted & overweight) in the same child needs to be considered. We found that overall 5.7% of the under-fives were stunted & overweight (HAZ < −2 and BAZ > +2), in comparison with 1.7% in the 5–9-year olds (HAZ < −2 and BAZ > +1). This prevalence is in the middle of the range of 0.3% to 10.9% reported by Tzioumis et al. [31] for 36 LMIC. Moreover, these researchers found that middle-income countries in actual fact had higher proportions of children who were stunted & overweight; at this point in time South Africa is classified as a upper-middle income country by the World Bank [43]. Our multivariate regression analysis in the total group showed that having a wealth index in quintile 3 protected against having astunted & overweight. Furthermore, the one-year olds in our study were significantly more likely to be stunted & overweight at 19.2%, with the prevalence decreasing to 5.8% in two-year olds, 1.7% in three-year olds and 0.1% in four-year olds. The protective effect of being older in terms of having being stunted & overweight was also evident from the multivariate regression analysis. Tzioumis et al. [31] surmised from available literature that one explanation for this dual burden of malnutrition in the same individual could be that poor early nutrition could result in preferential accumulation fat versus lean mass fostering a ‘thrifty phenotype’ with increased efficiency of fat storage. This is illustrated by research that showed that stunted children have a greater accumulation of fat mass and a lower lean mass gain when compared with their non-stunted counterparts. It follows that if stunted children overconsume energy relative to their needs, they may preferentially store it as fat, resulting in the dual burden of malnutrition [31].

The results of this study should be considered within the context of some limitations. Firstly, the study was only undertaken in two of the nine provinces in the country that are the most economically active and urbanized. Findings therefore do not reflect the situation in the country as a whole, but in our view make an important contribution to insights in the malnutrition patterns experienced by 1–<10-year old South African children. Secondly, comparisons with historical prevalence data for the country as a whole, as well as the two provinces, was challenging as a result of differences in sampling frames, sample sizes, age ranges and groupings, as well as malnutrition indicators and cut-offs used, especially for the identification of overweight and obesity. The latter is an internationally recognized problem [24]. De Onis and Lobstein [24] explain that BMI is the most practical, universally applicable, inexpensive and non-invasive anthropometric indicator for classifying overweight and obesity, for which reason BMI-for-age was used to identify wasting, overweight and obesity in this research. However, many of the older local and international studies and even more recent studies used for example WHZ for younger children and the IOTF BMI cut-points [41] for older children. Thirdly, it needs to be borne in mind that socio-demographic indicators were self-reported either by the biological mother or primary caregiver of the child.

## 5. Conclusions

Results of this cross-sectional survey of a representative sample of 1–<10-year old children in two South African provinces indicate that stunting and overweight/obesity are still present at concerning levels, especially in the under-five age group. Neither province nor living area emerged as predictors of any forms of malnutrition, although Chi-square comparison indicated that overweight in the under-five age group was higher in the WC than GTG. When compared to historical prevalence data there seems to be a trend towards reduced levels of stunting in the two provinces in both age groups. Overweight, but not obesity prevalence, seems to be on the decrease in the two provinces, especially in the WC. However, these apparent improvements may not be sufficient to achieve the SDG of ending all forms of malnutrition by 2030 [34], nor the 2015–2020 Strategy for Prevention and Control of Obesity of reducing obesity by 10% by 2020 [42].

Results showed consistently that those under two years of age were the most vulnerable to experiencing stunting, overweight/obesity and stunting & overweight. Given how critical the first 1000 days is for future growth and development our results emphasize that this age group should remain a key target population for nutrition intervention. It should, however, be borne in mind that interventions and approaches should be sensitive to the different forms of malnutrition as intervention needs may differ. For example, Tzioumis et al. [31] caution that interventions that target only stunting, especially those that promote ‘catch-up’ growth, may unintentionally contribute to overweight/obesity risk.

Associations found between indicators of socio-economic status investigated in this study and the different forms of malnutrition confirm that interventions that focus on addressing poverty and education remain crucial in the prevention/management of stunting. Although an obese mother was found to be protective of stunting, the fact of the matter is that interventions should rather focus on addressing household food security and child care and health within the context of also addressing the high levels of obesity amongst mothers of young children.

## Figures and Tables

**Table 1:** Cut-offs applied for the delineation of malnutrition in children aged 1–<10-years.

Age Group & Malnutrition Category [23,24]	HAZ	WAZ	BAZ
**1–<10-year olds (all children)**			
Stunted & very stunted	<−2 SD		
Very stunted	<−3 SD		
Underweight & severely underweight		<−2 SD	
Severely underweight		<−3 SD	
**1–<5-year olds only**			
Wasted			<−2 SD
At risk of overweight			> +1 SD; ≤ +2 SD
Overweight			> +2 SD; ≤ +3 SD
Obese			> +3 SD
Stunted & overweight	<−2 SD		> +2 SD
**5–<10-year olds only**			
Wasted			<−2 SD
Overweight			> +1 SD; ≤ +2 SD
Obese			> +3 SD
Stunted & overweight	<−2 SD		> +1 SD

**Table 2:** Demographic characteristics, wealth index quintiles, hunger scale scores and weight classification of mothers of 1–<10-year old children in Gauteng and Western Cape.

	Gauteng % (95% CI)	Western Cape % (95% CI)	Total Group % (95% CI)
**Who looks after the child most of the time**	N = 733	N = 592	N = 1325
Mother	70.2 (65.7–74.9) ##	71.0 (64.9–77.2)	70.5 (66.9–74.1)
Father	6.5 (3.4–9.6)	1.8 (0.2–3.3)	4.9 (2.8–7.0)
Grandparent	16.6 (12.8–20.4)	21.0 (15.5–26.5)	18.0 (15.0–21.1)
Other (sibling, aunt, uncle)	6.7 (4.0–9.5)	6.2 (2.4–10.1)	6.6 (4.4–8.8)
**Age of child**	N = 733	N = 593	N = 1326
1–<5 years	50.7 (46.4–55.0)	49.2 (42.1–56.3)	50.2 (46.5–53.8)
5–<10 years	49.3 (45.0–53.6)	50.8 (43.7–57.9)	49.8 (46.2–53.5)
**Gender**	N = 733	N = 593	N = 1326
Male	50.2 (45.6–54.9)	47.7 (43.2–52.2)	49.4 (46.0–52.8)
Female	49.8 (45.1–54.4)	52.3 (47.8–56.8)	50.6 (47.2–54.0)
**Head of household**	N = 733	N = 593	N = 1326
Father	40.2 (33.7–46.7) #	39.2 (34.8–43.6)	39.9 (35.5–44.4)
Mother	16.9 (13.9–19.9)	10.8 (7.0–14.6)	14.9 (12.4–17.1)
Grandmother	22.0 (15.5–28.4)	27.6 (21.0–34.1)	23.8 (19.0–28.5)
Grandfather	11.7 (8.5–15.0)	14.0 (10.3–17.8)	12.5 (10.1–15.2)
Other (e.g., aunt, uncle, friend)	9.2 (5.5–12.9)	8.4 (5.2–11.7)	9.0 (6.3–11.6)
**Marital status of mother**	N = 733	N = 593	N = 1326
Unmarried	41.2 (35.1–47.4) ###	34.3 (27.8–40.9)	39.0 (34.3–43.6)
Married	24.8 (20.3–29.4)	41.2 (33.3–49.0)	30.3 (26.3–34.2)
Divorced/separated/widowed	4.8 (2.5–7.0)	2.8 (0.7–4.9)	4.1 (2.5–5.7)
Living together	27.8 (22.0–33.5)	21.0 (16.2–25.9)	25.5 (21.4–29.6)
Other	1.4 (0.2–2.6)	0.7 (0.0–1.6)	1.1 (0.3–2.0)
**Mother’s highest education**	N = 733	N = 592	N = 1325
Less than grade 12	51.2 (44.9–57.5) ##	57.8 (47.2–68.4)	53.4 (48.0–58.8)
Grade 12	33.9 (28.3–39.5)	24.2 (17.1–31.3)	30.8 (26.3–35.0)
Qualification after grade 12	12.2 (8.6–15.8)	15.9 (7.8–24.1)	13.4 (9.9–17.0)
Do not know	2.7 (1.4–4.1)	2.1 (0.5–3.7)	2.5 (1.5–3.5)
**Father’s highest education**	N = 733	N = 592	N = 1325
Less than grade 12	26.9 (22.1–31.7)	33.6 (29.0–38.3)	29.1 (25.6–32.6)
Grade 12	32.8 (27.0–38.6)	29.7 (24.3–35.0)	31.8 (27.6–36.0)
Qualification after grade 12	13.2 (9.5–17.0)	11.0 (6.1–16.0)	12.5 (9.5–15.4)
Do not know	27.1 (22.1–32.1)	25.7 (20.2–31.1)	26.6 (22.9–30.4)
**Mother’s employment status**	N = 732	N = 593	N = 1325
Yes	22.3 (17.8–26.8) ###	39.2 (31.8–46.7)	28.0 (24.2–31.7)
No	74.6 (69.6–79.6)	59.4 (52.2–66.7)	69.6 (65.5–73.6)
Don’t know/not applicable	3.0 (1.3–4.8)	1.3 (0.3–2.4)	2.5 (1.3–3.7)
**Father’s employment status**	N = 731	N = 593	N = 1324
Yes	64.7 (60.4–69.0)	65.4 (59.7–71.1)	64.9 (61.5–68.3)
No	21.6 (17.7–25.6)	20.2 (14.8–25.7)	21.2 (18.0–24.3)
Don’t know/not applicable	13.7 (11.0–16.4)	14.4 (10.3–18.5)	13.9 (11.7–16.1)
**Wealth index quintiles**	N = 733	N = 593	N = 1326
One	21.1 (14.5–27.7) ##	17.3 (10.0–24.6)	19.8 (14.9–24.8)
Two	18.1 (12.3–23.9)	25.0 (20.8–29.1)	20.4 (16.4–24.4)
Three	21.0 (16.8–25.3)	16.0 (12.0–20.0)	19.4 (16.3–22.5)
Four	21.6 (16.9–26.4)	18.0 (12.8–23.1)	20.4 (16.9–23.9)
Five	18.1 (11.5–24.8)	23.7 (14.7–32.7)	20.0 (14.7–25.2)
**Ethnicity**	N = 732	N = 593	N = 1325
Black African	97.8 (96.0–99.7) ###	27.5 (13.0–41.9)	74.4 (69.6–79.3)
Mixed ancestry	2.1 (0.3–4.0)	68.1 (54.1–82.2)	24.1 (19.3–28.8)
Other	0.03 (0.0–0.07)	4.4 (0.6–8.2)	1.5 (0.2–2.7)
**Type of residence**	N = 733	N = 593	N = 1326
Rural	2.4 (2.4–2.5) ##	6.6 (5.7–7.5)	3.8 (3.5–4.1)
Urban formal	88.9 (88.7–89.1)	86.8 (85.7–87.9)	88.2 (87.8–88.6)
Urban informal	8.7 (8.5–8.9)	6.6 (6.2–7.0)	8.0 (7.8–8.2)
**Mother BMI**	N = 730	N = 513	N = 1243
Underweight/normal weight (BMI < 25)	33.2 (28.0–38.5) ##	28.9 (23.6–34.2)	31.9 (28.0–35.8)
Overweight (BMI ≥ 25; <30)	27.8 (23.6–31.9)	20.5 (16.5–24.6)	25.5 (22.4–28.6)
Obese (BMI ≥ 30)	38.9 (35.9–42.1)	50.6 (43.1–58.1)	42.6 (39.4–45.7)
**Hunger Scale [21]**	N = 733	N = 593	N = 1326
Total score = 0: No risk	58.1 (49.7–66.4) #	49.1 (39.7–58.6)	55.1 (48.8–61.4)
1–4: At risk of hunger	21.8 (17.1–26.6)	28.6 (22.9–34.3)	24.1 (20.4–27.8)
5–8: Food shortage in house	20.1 (14.9–25.3)	22.3 (16.8–27.8)	20.8 (16.9–24.7)

BAZ, body mass index-for-age-z scores; BMI, body mass index; 95% CI, 95% confidence intervals; # Significant relationship between the variable and province, Chi-square *p*-value < 0.05; ## *p* < 0.01; ### *p* < 0.001; N-values reflect actual number of cases, estimates are adjusted using relevant weighting.

**Table 3:** Prevalence of stunting, underweight, wasting, overweight, obesity and stunted & overweight in 1–<10-year old children in Gauteng and Western Cape by area of residence.

Parameter	Category	Gauteng 2018 (N= 723) Weighted % (95% CI)					Western Cape N = 577) Weighted% (95% CI)					Total Group (n = 1300) Weighted% (95% CI)
Area of residence		Urban Formal	Urban Informal	Rural	Chi Square *p*-Value	Total GTG	Urban Formal	Urban Informal	Rural	Chi Square *p*-Value	Total WC	All
Sample size	1–<5 years	N = 196	N = 87	N = 90		N = 373	N = 140	N = 79	N = 82		N = 301	N = 674
**Height-for-age**												
HAZ < −2 SD	Stunted	20.4 (13.2–27.7)	34.5 (27.3–41.7)	30.0 (18.8–41.2)	*p* > 0.05	22.0 (15.7–28.2)	21.4 (10.5–32.4)	11.4 (1.7–21.1)	23.2 (7.6–38.8)	*p* > 0.05	20.8 (11.6–30.0)	21.6 (16.5–26.7)
HAZ < −3 SD	Severely stunted	7.7 (1.7–13.6)	11.5 (5.8–17.2)	12.2 (3.0–21.5)	*p* > 0.05	8.1 (3.0–13.2)	4.3 (0.7–7.9)	5.1 (0.0–11.1)	6.1 (0.0–12.5)	*p* > 0.05	4.5 (1.4–7.5)	6.9 (3.4–10.5)
**Weight-for-age**												
WAZ < −2 SD	Underweight	5.1 (0.4–9.8)	8.0 (0.0–16.9)	12.2 (7.4–17.0_	*p* > 0.05	5.5 (1.4–9.7)	5.7 (1.2–10.2)	1.3 (0.0–4.1)	12.2 (1.8–22.6)	*p* > 0.05	5.8 (2.0–9.6)	5.6 (2.6–8.6)
WAZ < −3 SD	Severely underweight	0.5 (0.0–1.6)	2.3 (0.0–5.6)	-	*p* > 0.05	0.7 (0.0–1.6)	1.4 (0.0–3.4)	-	-	*p* > 0.05	1.2 (0.0–2.9)	0.9 (0.0–1.7)
**BMI-for-age**												
BAZ < −2 SD	Wasted	3.1 (0.0–6.5)	6.9 (3.2–10.6)	4.4 (0.0–9.9)	*p* > 0.05	3.5 (0.5–6.4)	2.1 (0.0–5.5)	-	3.7 (0.0–7.6)	*p* > 0.05	2.1 (0.0–4.9)	3.0 (0.8–5.2)
BAZ < −3 SD	Severely wasted	1.0 (0.0–2.4)	3.4 (0.0–7.2)	1.1 (0.0–3.6)	*p* > 0.05	1.3 (0–2.5)	0.7 (0.0–2.2)	-	-	*p* > 0.05	0.6 (0.0–1.9)	1.0 (0.1–2.0)
BAZ > +1 SD; ≤ + 2	At risk of overweight	19.4 (14.1–24.7)	20.7 (8.1–33.3)	27.8 (16.7–38.8)	*p* > 0.05	19.7 (15.0–24.4)	21.4 (11.6–31.2)	29.1 (17.2–41.0)	20.7 (10.7–30.8)	*p* > 0.05	22.0 (13.8–30.1)	20.4 (16.3–24.5)
BAZ > +2 SD; ≤ +3 SD	Overweight	7.7 (3.8–11.5)	11.5 (4.6–18.4)	8.9 (2.7–15.0)	*p* > 0.05	8.0# (4.7–11.4)	15.7 (8.6–22.8)	8.9 (2.3–15.4)	11.0 (1.3–22.6)	*p* > 0.05	14.9 (9.0–20.9)	10.3 (7.3–13.3)
BAZ > +3 SD;	Obese	6.6 (3.4–9.9)	8.0 (2.8–13.3)	8.9 (2.5–15.3)	*p* > 0.05	6.8 (4.0–9.6)	7.1 (3.9–10.4)	12.7 (3.6–21.7)	4.9 (1.2–8.6)	*p* > 0.05	7.4 (4.7–10.2)	7.0 (4.9–9.1)
**Height-for-age and BMI-for-age (combined)**												
HAZ < −2 SD and BAZ > +2 SD	Stunted & overweight	5.6 (3.0–8.2)	6.9 (0.0–13.9)	8.9 (4.0–13.8)	*p* > 0.05	5.8 (3.5–8.2)	5.7 (1.0–10.4)	2.5 (0.0–8.2)	4.9 (0.0–11.0)	*p* > 0.05	5.4 (1.5–9.4)	5.7 (3.7–7.7)
**Mid-upper arm circumference**												
MUACZ < −2 SD	Moderate undernutrition	3.1 (0.8–5.3)	-	3.3 (0.0–7.0)	*p* > 0.05	2.8 (0.9–4.7)	2.2 (0.0–4.8)	1.3 (0.0–4.4)	6.3 (0.0–13.9)	*p* > 0.05	2.4 (0.1–4.6)	2.7 (1.2–4.1)
MUACZ < −3 SD	Severe undernutrition	1.5 (0.0–3.3)	-	2.2 (0.0–5.5)	*p* > 0.05	1.4 (0.0–2.9)	1.4 (0.0–3.7)	-	2.5 (0.0–7.6)	*p* > 0.05	1.4 (0.0–3.3)	1.4 (0.2–2.6)
**Sample size**	**5–<10 years**	**N = 196**	**N = 71**	**N = 83**		**N = 350**	**N = 133**	**N = 70**	**N = 73**		**N = 276**	**N = 626**
**Height-for-age**												
HAZ < −2 SD	Stunted	6.6 (3.0–10.3)	7.0 (0.0–15.5)	12.0 (4.4–19.7)	*p* > 0.05	6.8 (3.6–10.0)	6.0 (2.2–9.9)	5.7 (0.7–10.8)	15.1 (2.5–27.6)	*p* > 0.05	6.6 (3.2–10.0)	6.7 (4.4–9.1)
HAZ < −3 SD	Severely stunted	2.6 (0.0–5.6)	1.4 (0.0–4.5)	3.6 (0.0–9.2)	*p* > 0.05	2.5 (0.0–5.2)	1.5 (0.0–3.7)	-	-	*p* > 0.05	1.3 (0.0–3.1)	2.1 (0.2–3.9)
**Weight-for-age**												
WAZ < −2 SD	Underweight	5.1 (1.8–8.4)	5.6 (0.6–10.7)	6.0 (0.0–12.0)	*p* > 0.05	5.2 (2.2–8.1)	3.0 (0.0–6.7)	7.1 (0.0–14.4)	5.5 (0.4–10.5)	*p* > 0.05	3.4 (0.2–6.6)	4.6 (2.4–6.8)
WAZ < −3 SD	Severely underweight	1.5 (1.8–8.4)	4.2 (0.0–9.2)	-	*p* > 0.05	1.7 (0.0–3.8)	-	1.4 (0.0–4.7)	1.4 (0.0–4.5)	*p* > 0.05	0.2 (0.0–0.4)	1.2 (0.0–2.5)
**BMI-for-age**												
BAZ < −2 SD	Wasted	7.1 (2.8–11.5)	5.6 (0.2–11.1	2.4 (0.0–6.1)	*p* > 0.05	6.9 (3.0–10.8)	3.0 (0.1–5.9)	4.3 (0.0–9.0)	5.5 (0.0–12.4)	*p* > 0.05	3.3 (0.8–57)	5.7 (3.0–8.3)
BAZ < −3 SD	Severely wasted	2.6 (0.0–6.0)	2.8 (0.0–7.1)	1.2 (0.0–3.9)	*p* > 0.05	2.5 (0.0–5.5)	1.5 (0.0–3.7)	1.4 (0.0–4.7)	-	*p* > 0.05	1.4 (0.0–3.2)	2.2 (0.1–4.2)
BAZ > +1 SD; ≤ + 2	Overweight	12.2 (7.2–17.2)	12.7 (3.8–21.5)	12.0 (4.1–20.0)	*p* > 0.05	12.3 (7.9–16.7)	16.5 (10.2–22.9)	14.3 (3.5–25.1)	6.8 (2.5–11.2)	*p* > 0.05	15.7 (10.4–21.1)	13.4 (10.1–16.8)
BAZ > +2 SD	Obese	8.2 (3.6–12.7)	1.4 (0.0–4.6)	2.4 (0.0–5.9)	*p* > 0.05	7.5 (3.5–11.4)	5.3 (1.0–9.5)	7.1 (1.7–12.6)	8.2 (1.1–15.4)	*p* > 0.05	5.6 (1.9–9.2)	6.8 (4.0–9.7)
**Height-for-age and BMI-for-age (combined)**												
HAZ < −2 SD and BAZ > +1 SD	Stunted & overweight	2.0 (0.0–4.0)	-	4.8 (0.0–10.6)	*p* > 0.05	1.9 (0.2–3.7)	1.5 (0.0–3.7)	-	-	*p* > 0.05	1.3 (0.0–3.2)	1.7 (0.4–3.0)

HAZ, height-for-age z-scores; WAZ, weight-for-age z-scores; BAZ, body mass index-for-age z-scores; MUACZ, mid-upper arm circumference z-scores; 95% CI, 95% confidence intervals. All values in table are representative of the total population (12–119 months) unless otherwise noted. N-values reflect actual number of cases, estimates are adjusted using relevant weighting. # Significant relationship between province and overweight for children <60 months, *p* < 0.05, Chi square test.

**Table 4:** Prevalence of stunting, underweight, overweight, obesity and stunted & overweight in children aged 1–<10-years in Gauteng and Western Cape by year age groups.

Parameter	Category	Gauteng and Western Cape 2018 (n = 1300) % (95% CI)								
Age Group		1 year 12–<24 Mths	2 years 24–<36 Mths	3 years 36–<48 Mths	4 years 48–<60 Mths	5 years 60–<72 Mths	6 years 72–<84 Mths	7 years 84–<96 Mths	8 years 96–<108 Mths	9 years 108–<120 Mths
Sample size	Description	N = 144	N = 175	N= 197	N=158	N = 152	N = 152	N = 126	N = 121	N = 75
**Height-for-age**										
HAZ < −2 SD	Stunted	39.0 (27.2–50.9)	23.2 (14.0–32.5)	17.2 (9.7–24.7)	11.9 (5.0–18.8)	7.5 (1.7–13.3)	5.3 ^c^ (1.3–9.4)	4.2 (0.0–8.7)	9.8 (3.4–16.2)	7.2 (0.0–14.6)
HAZ < −3 SD	Severely stunted	14.9 (7.8–22.1)	7.2 (1.9–12.6)	5.7 ^1^ (0.6–10.8)	2.1 (0.0–4.6)	1.3 (0.0–3.2)	2.1 (0.0–5.0)	1.7 (0.0–4.9)	3.3 (0.0–7.5)	2.4 (0.0–7.1)
**BMI-for-age**										
BAZ < −2 SD	Wasted	1.5 (0.0–3.8)	5.8 (0.0–11.7)	1.8 (0.0–4.2)	2.6 (0.0–5.2)	4.0 (0.2–7.8)	9.8 (1.9–17.6)	1.8 (0.0–4.3)	6.9 (1.0–12.9)	4.7 (0.0–10.9)
BAZ < −3 SD	Severely wasted	1.4 (0.0–3.7)	1.4 (0.0–3.8)	1.0 (0.0–3.1)	0.4 (0.0–1.0)	0.3 (0.0–0.9)	4.9 (0.0–12.1)	1.3 (0.0–3.8)	3.2 ^d^ (0.0–7.3)	-
BAZ > +1 SD; ≤ +2	At risk of overweight (1–<5 years)	30.6 (22.7–38.5)	23.3 (15.0–31.6)	19.8 (11.6–28.1)	10.5 (4.0–17.0)					
BAZ > +1 SD; ≤+2	Overweight (5–<10 years)					17.7 (8.9–26.6)	10.1 (3.9–16.4)	13.2 (5.8–20.6)	12.3 (5.1–19.4)	13.66^3^ (3.9–23.3)
BAZ >+2 SD, ≤3	Overweight (1–<5 years)	23.3 ^a^ (15.1–31.5)	11.2 (4.7–17.7)	6.6 ^2^ (2.5–10.6)	3.9 (0.5–7.3)					-
BAZ >+2 SD	Obese (5–<10 years)					6.4 (1.9–11.0)	7.6 (2.8–12.3)	2.0 (0.0–5.2)	8.2 (0.8–15.6)	11.2 ^e^ (1.8–20.7)
BAZ > +3 SD;	Obese (1–<5 years)	14.2 (7.1–21.3)	9.1 (3.1–15.0)	3.2 (0.2–6.1)	4.0 (0.4–7.5)					
**Height-for-age and BMI-for age combined**										
HAZ < −2 SD and BAZ > +2 SD	Stunted & overweight (1–<5 years)	19.2 ^b^ (11.1–27.3)	5.8 (1.6–10.0)	1.7 (0.0–3.7)	0.1 (0.0–0.2)					
HAZ < −2 SD and BAZ > +1 SD	Stunted & overweight (5–<10 years)					2.2 (0.0–5.1)	1.3 (0.0–3.6)	0.2 (0.0–0.5)	3.2 (0.0–7.4)	1.7 (0.0–5.3)

HAZ, height-for-age z-scores, BAZ, body mass index-for-age z-scores; 95% CI, 95% confidence intervals. All values in table are representative of the total population (1–<10 years) N-values reflect actual number of cases, estimates are adjusted using relevant weighting 1: Age = 3, HAZ < −3 SD: Gauteng = 9.0%, WC = 0.2%, Chi square *p* < 0.05; 2: Age = 3, BAZ > +2 SD, ≤3 SD: Gauteng = 2.6%, WC = 13.2%, Chi square *p* < 0.05; 3: Age = 9, BAZ > +1 SD; ≤ +2, Gauteng = 5.3%, WC = 24.2%, Chi square *p* < 0.05 a: Age = 1, BAZ > +2 SD, ≤3SD, Female = 31.4%, Male = 13.3%, Chi square *p* < 0.05; Chi square *p* < 0.05; b: Age = 1, HAZ < −2 SD and BAZ > +2 SD, Female = 26.4%, Male = 10.3%, Chi square *p* < 0.05, c: Age = 6, HAZ < −2 SD, Female = 0.3%, Male = 11.0%, Chi square < 0.01, d: Age = 8, BAZ < −3 SD, Female = 0.0%, Male = 7.5%, Chi square *p* < 0.05; e: Age = 9: BAZ > +2 SD, Female = 17.3%, Male = 0.7%, Chi square *p* < 0.05.

**Table 5:** Bivariate logistic regression analysis to identify socio-demographic predictors of stunting, at risk of overweight or overweight or obese and combination of stunting & overweight in 1–<10-year old children in Gauteng and Western Cape.

	Children 1–<10 years Stunted N = 1300 (n = 206) Odds Ratio (95% CI)	Children 1–<10 years At Risk of Overweight/Overweight/Obese (BAZ > +1 SD) N = 1300 (n = 391) Odds Ratio (95% CI)	Children 1–<10 years Stunted & Overweight (HAZ < −2 SD & BAZ > +2 SD for 1-< 5 year olds or >=1 SD for 5-<10 year olds) N = 1300 (n = 86) Odds Ratio (95% CI)
**Who mostly looks after the child**			
Mother	Ref	Ref	Ref
Father	1.20 (0.53–2.44)	0.79 (0.40–1.47)	1.78 (0.61–4.26)
Grandparent	1.08 (0.68–1.68)	1.02 (0.72–1.44)	0.99 (0.47–1.90)
Other (sibling, aunt, uncle, etc.)	1.94 (0.42–6.55)	1.07 (0.28–3.39)	3.37 (0.54–12.91)
**Age in years**			N = 1300
1–<5-years	3.77 (2.56–5.68) ***	2.38 (1.81–3.14) ***	5.97 (3.10–12.78) ***
5–<10-years	Ref	Ref	Ref
**Gender**			
Male	Ref	Ref	Ref
Female	0.90 (0.64–1.28)	0.93 (0.71–1.21)	1.00 (0.60–1.67)
**Head of household**			
Father	Ref	Ref	Ref
Mother	0.97 (0.54–1.66)	0.81 (0.53–1.22)	1.21 (0.54–2.51)
Grandmother	1.32 (0.84–2.06)	0.88 (0.62–1.25)	0.93 (0.45–1.86)
Grandfather	1.49 (0.86–2.51)	1.31 (0.87–1.98)	1.22 (0.51–2.64)
Other (e.g., aunt, uncle, friend, cousin)	1.56 (0.84–2.77)	1.01 (0.62–1.63)	1.86 (0.80–4.01)
**Marital status of mother**			
Unmarried	Ref	Ref	Ref
Married	0.71 (0.45–1.12)	1.03 (0.75–1.42)	1.04 (0.56–1.92)
Divorced/separated/widowed	1.17 (0.47–2.57)	0.66 (0.29–1.34)	0.18 (0.00–1.32)
Living together	1.38 (0.91–2.08)	1.07 (0.77–1.50)	1.09 (0.57–2.04)
Other	1.14 (0.18–4.31)	1.51 (0.45–4.59)	3.01 (0.47–11.79)
**Mother’s highest level of education**			
Less than grade 12	Ref	Ref	Ref
Grade 12	0.72 (0.48–1.05)	1.27 (0.94–1.71)	1.18 (0.67–2.05)
Qualification after grade 12	0.38 (0.18–0.71) **	1.14 (0.76–1.70)	0.59 (0.20–1.40)
Do not know	0.47 (0.09–1.49)	0.94 (0.37–2.15)	1.36 (0.24–4.66)
**Father’s highest level of education**			
Less than grade 12	Ref	Ref	Ref
Grade 12	0.76 (0.48–1.20)	1.21 (0.85–1.71)	0.90 (0.45–1.80)
Qualification after grade 12	0.68 (0.35–1.25)	1.29 (0.82–2.01)	1.03 (0.41–2.39)
Do not know	1.34 (0.87–2.07)	1.23 (0.86–1.77)	1.32 (0.69–2.58)
**Mother’s employment status**			
Yes	Ref	Ref	Ref
No	1.29 (0.88–1.93)	1.03 (0.77–1.39)	1.32 (0.75–2.47)
Don’t know/not applicable	0.51 (0.01–3.50)	0.17 (0.00–1.18)	1.01 (0.01–8.65)
**Father’s employment status (%)**			
Yes	Ref	Ref	Ref
No	1.02 (0.66–1.55)	0.93 (0.67–1.30)	0.88 (0.43–1.64)
Don’t know/not applicable	1.16 (0.62–2.04)	0.97 (0.59–1.54)	1.08 (0.41–2.41)
**Wealth index quintiles**			
One	Ref	Ref	Ref
Two	0.90 (0.55–1.48)	0.79 (0.52–1.20)	1.01 (0.46–2.23)
Three	0.58 (0.33–1.00)	0.63 (0.40–0.97) *	0.67 (0.27–1.60)
Four	0.53 (0.30–0.92) *	0.80 (0.52–1.21)	1.02 (0.46–2.53)
Five	0.63 (0.37–1.07)	1.25 (0.83–1.87)	1.11 (0.51–2.43)
**Ethnicity**			
Black African	Ref	Ref	Ref
Mixed ancestry	1.41 (0.95–2.05)	0.84 (0.61–1.15)	0.90 (0.47–1.62)
Other	0.27 (0.01–1.69)	1.05 (0.34–2.87)	0.63 (0.01–4.04)
**Province**			
Gauteng	1.07 (0.74–1.56)	0.77 (0.59–1.02)	1.17 (0.68–2.09)
Western Cape	Ref	Ref	Ref
**Type of residence**			
Rural	Ref	Ref	Ref
Urban formal	0.61 (0.29–1.42)	1.07 (0.54–2.23)	0.60 (0.23–2.14)
Urban informal	0.90 (0.36–2.38)	1.18 (0.53–2.77)	0.71 (0.19–3.04)
**Mother’s BMI**			
Underweight/normal weight	Ref	Ref	Ref
Overweight	0.84 (0.55–1.27)	0.99 (0.69–1.40)	0.87 (0.46–1.61)
Obese	0.42 (0.28–0.64) ***	1.16 (0.85–1.58)	0.55 (0.30–1.01)
**Hunger scale**			
Total score=0: No risk	Ref	Ref	Ref
1–4: At risk of hunger	1.03 (0.66–1.57)	0.87 (0.63–1.21)	0.92 (0.48–1.68)
5–8: Food shortage in house	1.34 (0.87–2.04)	0.74 (0.52–1.05)	0.82 (0.39–1.57)

BAZ, body mass index-for-age-z scores; BMI, body mass index; * Odds ratio significant, *p* < 0.05; ** Odds ratio significant, *p* < 0.01; *** Odds ratio significant, *p* < 0.001; N-values reflect the total number of children, n represents the number of children in the risk group, estimates are adjusted using relevant weighting.

**Table 6:** Multivariate logistic regression analysis to identify socio-demographic predictors of stunting, at risk of overweight or overweight or obesity and combination of stunting & overweight in 1–<10-year old children in Gauteng and Western Cape.

	1–<10 years Stunted (HAZ < −2 SD) N = 1300 (n = 206) OR (95% CI)	1–<10 years At risk of Overweight/Overweight/Obese (BAZ > +1 SD) N = 1300 (n = 391) OR (95% CI)	1–<10 years Stunted & Overweight (HAZ < −2 SD & BAZ > +2 SD for 1-< 5 year olds or >=1 SD for 5-<10 year olds) N = 1300 (n = 86) OR (95% CI)
**Age in years**			
1–<5-years	3.73 (2.52–5.66)	2.39 (1.81–3.16) ***	5.97 (3.10–12.78) ***
5–<10-years	Ref	Ref	Ref
**Mother’s highest level of education**			
Less than grade 12	Ref		
Grade 12	0.68 (0.44–1.03)		
Qualification after grade 12	0.34 (0.16–0.68) **		
Do not know	0.68 (0.13–2.29)		
**Wealth index quintiles**			
One	Ref	Ref	
Two	1.09 (0.65–1.83)	0.80 (0.52–1.23)	
Three	0.66 (0.37–1.16)	0.62 (0.40–0.97) *	
Four	0.79 (0.43–1.41)	0.83 (0.54–1.27)	
Five	1.02 (0.56–1.84)	1.28 (0.85–1.94)	
**Mother’s BMI**			
Underweight or Normal weight	Ref		
Overweight	0.86 (0.56–1.33)		
Obese	0.46 (0.30–0.71) **		

BAZ, body mass index-for-age-z scores; BMI, body mass index; * Odds ratio significant, *p* < 0.05; ** Odds ratio significant, *p* < 0.01; *** Odds ratio significant, *p* < 0.001 N-values reflect the total number of children, n represents the number of children in the risk group, estimates are adjusted using relevant weighting.

**Table 7:** Overview of malnutrition (stunting, underweight, wasting, at risk of overweight, overweight and obesity) ^a^ prevalence in South African children under−10 years old as recorded in national surveys compared to data recorded in the Provincial Dietary Intake Study (PDIS).

Indicator and Cut-Offs	1993 SAVACG N = 10819 6–71 Mths Olds [13]		NFCS 1999 N = 2894 1–9 Years Olds [15]			2003 SAHDS N = 1159 0–<5 Years Olds [29]	2005 NFCS- FB−1 N = 1099 1–<5 Years Olds [29]	2008 NIDS N = 4268 6 Mths−9 Years Olds [30]		2012 SANHANES N = 1416 <5 Years Olds [29]	2016 SADHS N = 1416 6 mnths−5 Years Olds [7]	2018 PDIS: Current Study N = 1300 1–9 Years Olds		
Age group (years except if otherwise indicated)	12–23 mths N = 2027	6–71 mths N = 10819	1–3 y	4–6 y	7–9 y	0–<5 y	1–<5 y	6 mths−4 y	5–9 y	1–< 5 y	<5 y	1 y N = 144	1–<5 y N = 674	5<10 y N = 626
**Stunted, underweight or wasted**														
Stunted (all): HAZ < −2 SD (%)	23.4	16.1	24.4	19.7	11.3	33.4	26.4	24.9	12.2	26.5	27.4	39.0	21.6	6.7
Underweight (all): WAZ < −2 SD (%)	9.0	9.3	11.4	8.0	6.4	9.9	6.3			6.2	5.9	1.0	5.6	4.6
Wasted < 5 y olds: WHZ < −2 SD (%)	3.9	2.6	3.4	3.3	3.2	7.5	6.0			2.9	2.5			
**At risk of overweight, overweight or obese**														
At risk overweight < 5 y olds; or overweight 5–9 y olds: WHZ > 1; ≤2 SD (%)			13.4	13.4	9.5	20.1	16.6			22.8				
Overweight < 5 y olds: WHZ > 2; ≤3 SD (%)						11.3	6.8			10.7				
Overweight/obese < 5 olds; or Obese 5–9 y olds: WHZ > 2 SD (%)			7.4	5.9	6.5						13.3			
Obese < 5 y olds WHZ ≥ 3 %						7.0	1.6			3.5				
Wasting (All): BAZ <−2 SD (%)						7.1	6.3			2.6		1.5	3.0	5.7
At risk overweight < 5 y olds: BAZ > 1; ≤2 SD (%)						20.7	7.6			24.9		30.6	20.4	
Overweight for <5 y olds: BAZ > 2; ≤3 SD (%)						12.0	10.2	16.1		12.0		23.3	10.3	
Obese < 5 y olds BAZ ≥ 3 (%)						8.5	2.6	4.8		4.0		14.2	7.0	
Overweight for 5–9 y olds: BAZ > 1; ≤2 SD (%)									18.2					13.4
Obese 5–9 y olds BAZ > 2 (%)									7.5					6.8
Overweight IOTF^8^ BMI ≥ 25; <30 kg/m^2^ (%)			16.0	12.0	6.5									
Obese IOTF^8^ ≥30 kg/m^2^ (%)			6.1	2.5	1.1									

Mths, months; Y, year; GTG, Gauteng; WC, Western Cape; HAZ, height-for-age z-scores; WAZ, weight-for-age z-scores; BAZ, body mass index-for-age z-scores; MUACZ, mid-upper arm circumference z-scores; SD, Standard Deviation ^a^ HAZ, WAZ, WHZ and BAZ indicators from historical surveys were included in this table although WHZ was not reported on in the PDIS. Note that where both the WHZ and BAZ were reported, prevalence of wasting, at risk of overweight, overweight and obesity was very similar.

## Supplementary Materials

The following are available online at https://www.mdpi.com/1660-4601/16/18/3334/s1, Table S1: Loading of household possessions included in the wealth index for 5 quintiles, Table S2: Bivariate logistic regression analysis to identify individual wealth index item predictors of stunting, at risk of overweight or overweight or obesity and combination of stunting & overweight in 1-< 10 -year old children in Gauteng and Western Cape.
