# Supplementary material for: Provincial Dietary Intake Study (PDIS): Prevalence and Sociodemographic Determinants of the Double Burden of Malnutrition in A Representative Sample of 1 to Under 10-Year-Old Children from Two Urbanized and Economically Active Provinces in South Africa
Source: Int J Environ Res Public Health. 2019 Sep 10;16(18):3334. doi: 10.3390/ijerph16183334 (PMC6765782; doi:10.3390/ijerph16183334)
Supplement: Supplementary file 1 [file ijerph-16-03334-s001.zip › ijerph-582563-supple-for xml.pdf]

Supplementary Table S1. Loading of household possessions included in the wealth index for 5 quintiles

Household Possessions (%)	Gauteng **Wt% (95% CI)** (N=733)	Western Cape **Wt% (95% CI)** (N=593)	ALL **Wt% (95% CI)** (N=1326)	Asset group 1 Wt% (20.0%)	Asset group 2 Wt% (20.0%)	Asset group 3 Wt% (19.5%)	Asset group 4 Wt% (20.5%)	Asset group 5 Wt% (20.0%)
Refrigerator	82.7 (78.4-86.9)	85.3 (79.2-91.3)	83.5 (80.1-86.9)	42.9###	75.5	99.2	100.0	100.0
Stove (any type)	92.8 (88.6-97.0)	95.0 (92.7-97.4)	93.5 (90.7-96.4)	69.1###	98.4	100.0	100.0	100.0
Microwave	54.3 (46.9-61.6)###	68.1 (59.8-76.3)	58.8 (53.3-64.4)	19.3###	48.2	39.0	87.0	99.3
Washing machine	41.3 (32.8-49.8)###	66.4 (57.5-75.4)	49.6 (43.3-56.0)	13.7###	39.8	28.5	68.1	97.0
Vacuum cleaner	5.2 (1.6-8.9)###	22.8 (15.7-29.8)	11.1 (7.8-14.4)	2.0###	6.3	8.9	11.3	26.8
Radio	60.9 (55.7-66.1)	56.5 (49.0-64.0)	59.4 (55.2-63.6)	43.9###	50.3	59.1	70.3	73.4
Television	88.4 (84.7-92.0)	91.3 (87.1-95.4)	89.3 (86.6-92.1)	55.3###	91.1	100.0	100.0	100.0
Computer	17.8 (12.2-23.4)###	29.6 (21.2-38.0)	21.7 (17.1-26.3)	5.9###	14.1	12.3	19.9	56.2
Landline telephone	0.5 (0.0-1.1)###	11.0 (5.1-16.9)	4.0 (2.1-5.9)	0.0###	4.6	0.6	2.6	12.0
Watch	45.7 (38.3-53.2)###	63.4 (55.2-71.5)	51.6 (46.0-57.2)	30.5###	41.5	42.0	56.3	87.4
Cell phone	97.0 (95.2-98.8)	95.5 (93.0-97.9)	96.4 (95.1-97.9)	89.6###	96.3	98.5	98.5	99.5
Bicycle	15.7 (11.8-19.6)###	32.0 (23.5-40.5)	21.1 (17.3-24.9)	6.6###	21.0	11.1	18.1	48.6
Motorcycle	3.6 (1.7-5.6)###	11.1 (7.0 – 15.1)	6.1 (4.3-7.9)	1.3###	6.6	3.5	5.0	14.1
Car	27.1 (20.8-33.5)###	40.1 (30.9-49.3)	31.4 (26.3-36.6)	9.2###	19.3	17.9	32.9	77.6
Have electricity	93.9 (89.7-98.0)###	99.2 (98.3-100.0)	95.7 (92.9-98.4)	78.1###	100.0	100.0	100.0	100.0
Main cooking fuel (%)	N=732	N=588	1320					
Electric	91.7 (87.5-96.0)###	84.0 (78.6-89.3)	89.2 (85.9-92.5)	65.0###	81.9	98.8	100.0	100.0
Gas	1.7 (0.5-2.9)	14.7 (9.5-19.8)	6.0 (4.1-7.9)	10.6	18.1	1.2	0.0	0.0
Paraffin	6.1 (2.5-9.8)	0.2 (0.0-0.3)	4.2 (1.8-6.5)	21.0	0.0	0.0	0.0	0.0
Wood/coal	0.4 (0.0-1.0)	1.2 (0.1-2.2)	0.7 (0.2-1.2)	3.4	0.0	0.0	0.0	0.0
Type of toilet (%)	N=733	N=591	1324					
Flush toilet in house	57.5 (45.5-69.5)###	80.2 (73.0-87.3)	65.0 (56.7-73.3)	23.3###	55.9	74.1	80.0	91.6
Pit latrine	13.7 (5.4-22.0)	0.5 (0.1-0.8)	9.3 (3.8-14.8)	30.0	10.7	3.3	1.5	1.2
Bucket	0.6 (0.0-1.3)	2.4 (0.6-4.1)	1.2 0.5-1.9)	4.8	0.7	0.6	0.0	0.0
Chemical toilet	4.3 (2.2-6.3)	1.3 (0.0-3.8)	3.3 (1.7-4.9)	13.5	2.3	0.0	0.0	0.7
No facility	0.6 (0.0-1.5)	0.1 (0.0-0.3)	0.5 (0.0-1.1)	1.0	0.8	0.5	0.0	0.0
Other (flush outside)	23.3 (13.0-33.7)	15.5 (8.7-22.4)	20.8 (13.6-27.9)	27.5	29.7	21.5	18.6	6.4
Type of drinking water (%)	N=733	N=593	1326					
Tap in house	45.3 (35.1-55.5)###	79.3 (73.5-85.1)	56.6 (49.5-63.6)	24.4###	48.8	61.3	72.3	75.9
Tap in yard	45.6 (36.3-54.9)	8.6 (4.3-12.8)	33.3 (27.0-39.6)	38.1	47.4	36.8	26.1	18.0
Communal tap	8.8 (2.9-14.7)	4.5 (1.0-8.1)	7.4 (3.3-11.4)	36.1	1.0	0.0	0.0	0.0
Bottled water	0.3 (0.0 – 0.8)	7.6 (2.8-12.3)	2.7 (1.2-4.3)	1.3	2.8	1.9	1.6	6.2
Other (e.g. river/dam)	-	0.1 (0.0-0.1)	0.02 (0.0-0.04)	0.1	0.0	0.0	0.0	0.0
Type of dwelling (%)	N=733	N=593	1326					
Brick house/flat	72.3 (63.0-81.7)###	79.7 (73.8-85.6)	74.8 (68.3-81.2)	24.7###	55.8	93.5	99.9	100.0
Informal structure (shack/tin)	26.8 (17.6-36.0)	10.9 (5.1-13.7)	21.5 (15.3-27.8)	69.0	36.7	1.9	0.0	0.0
Other (mud/wooden/other)	0.8 (0.0-1.8)	9.4 (6.4-15.5)	3.7 (2.1-5.3)	6.3	7.5	4.6	0.1	0.0
Mean number rooms in house (95% CI)	2.9 (2.6 – 3.1)	2.9 (2.6 – 3.3)	2.9 (2.7 – 3.1)	2.0 (1.8 - 2.2) [E]	2.4 (2.1 - 2.6) [D]	2.7 (2.4 – 3.0) [C]	3.2 (2.9 – 3.4) [B]	4.2 (3.9 – 4.5) [A]
Mean number sleeping in house (95% CI)	5.2 (4.9 – 5.5)&&&	5.8 (5.5 – 6.2)	5.4 (5.2 – 5.6)	4.8 (4.4-5.1) [C]	5.2 (4.8-5.7) [B][C]	5.3 (5.0 – 5.7) [B]	5.8 (5.5 – 6.4) [A][B]	5.9 (5.4 – 6.2) [A]

Mean wealth index (95% CI)	0.5 (-0.2 – 1.2)&&&	1.3 (0.9 – 1.7)	0.8 (0.3 – 1.2)	-4.3 (-5.9- -2.8) [D]	0.6 (0.6 – 0.7) [C]	1.9 (1.9 – 1.9) [B]	2.5 (2.5 – 2.5) [A]	3.0 (3.0 – 3.0) [A]

###Significant relationship between the variable and the province / wealth index category, Chi-square p-value<0.0001
[A], [B], [C], [D], [E]: significant difference between the mean values for different wealth index groups, Bonferroni multiple comparison, p<0.05
&&&Significant difference between two provinces, independent t-test, p<0.0001
N-values reflect actual number of cases, estimates are adjusted using relevant weighting

Supplementary Table S2. Bivariate logistic regression analysis to identify individual wealth index item predictors of stunting, at risk of overweight or overweight or obesity and combination of stunting & overweight in 1-< 10 -year old children in Gauteng and Western Cape

Have Household Possessions (%)	**Stunted (HAZ < -2SD) N=1300 (n=206)**	**At risk of overweight/ Overweight/obese (BAZ > +1SD) N=1300 (n=391)**	**Stunted and at overweight (HAZ < −2 SD & BAZ > +21 SD for 1-< 5 year olds or >=1 SD for 5-<10 year olds) N=1300 (n=86)**
Refrigerator	0.52 (0.35-0.79)**	0.91 (0.64-1.31)	0.58 (0.32-1.09)
Stove (any type)	0.60 (0.33-1.16)	0.96 (0.56-1.71)	1.29 (0.47-5.27)
Microwave	0.51 (0.36-0.72)**	1.30 (0.99-1.71)	0.74 (0.44-1.24)
Washing machine	0.75 (0.53-1.07)	1.13 (0.86-1.48)	0.94 (0.56-1.57)
Vacuum cleaner	0.34 (0.14-0.70)**	1.34 (0.88-2.01)	0.50 (0.15-1.28)
Radio	0.92 (0.65-1.31)	0.94 (0.72-1.24)	0.70 (0.42-1.18)
Television	0.59 (0.36-0.99)*	0.98 (0.64-1.53)	1.25 (0.55-3.48)
Computer	0.65 (0.40-1.02)	1.28 (0.93-1.76)	0.69 (0.33-1.31)
Landline telephone	0.66 (0.20-1.64)	1.58 (0.82-2.94)	0.80 (0.13-2.63)
Watch	0.90 (0.64-1.28)	1.01 (0.77-1.32)	0.79 (0.47-1.32)
Cell phone	0.40 (0.20-0.87)*	0.71 (0.37-1.44)	0.42 (0.17-1.27)
Bicycle	0.85 (0.54-1.30)	1.09 (0.78-1.50)	0.84 (0.42-1.57)
Motorcycle	0.57 (0.22-1.26)	1.55 (0.91-2.58)	0.58 (0.11-1.77)
Car	0.74 (0.49-1.08)	1.29 (0.97-1.71)	1.39 (0.81-2.34)
Electricity	0.37 (0.20-0.75)**	0.81 (0.44-1.57)	0.53 (0.22-1.65)
Main cooking fuel (%)			
Electric	Ref	Ref	Ref
Gas	0.78 (0.32-1.65)	1.11 (0.63-1.91)	0.73 (0.17-2.20)
Paraffin	2.38 (1.13-4.70)*	0.95 (0.46-1.85)	1.07 (0.23-3.18)
Wood/coal/other	4.15 (0.87-17.08)	0.26 (0.01-1.68)	1.31 (0.02-9.51)
Type of toilet (%)			
Flush toilet in house	Ref	Ref	Ref
Pit latrine	1.34 (0.73-2.31)	0.83 (0.50-1.33)	0.56 (0.16-1.45)
Bucket	4.03 (1.23-12.10)*	0.58 (0.12-1.98)	1.97 (0.25-8.05)
Chemical toilet	1.62 (0.64-3.59)	1.20 (0.57-2.41)	1.84 (0.54-4.84)
No facility	0.74 (0.01-6.10)	0.86 (0.09-4.81)	1.31 (0.01-11.98)
Other (flush outside)	0.94 (0.59-1.47)	1.01 (0.72-1.41)	0.58 (0.26-1.15)
Type of drinking water (%)			

Tap in house	Ref	Ref	Ref
Tap in yard	1.38 (0.95-2.00)	0.84 (0.62-1.12)	0.85 (0.47-1.49)
Communal tap	1.77 (0.94-3.16)	1.18 (0.70-1.95)	1.43 (0.56-3.20)
Other (Bottled water/river)	1.21 (0.37-3.15)	1.33 (0.59-2.86)	0.97 (0.13-3.63)
Type of dwelling (%)			
Brick house/flat	Ref	Ref	Ref
Informal structure (shack/tin)	1.63 (1.09-2.40)*	0.97 (0.69-1.34)	0.90 (0.45-1.66)
Other (mud/wooden/other)	2.01 (0.85-4.30)	0.95 (0.44-1.92)	1.58 (0.41-4.38)
Mean number rooms in house (95% CI)	0.88 (0.78-1.00)	1.00 (0.91-1.10)	1.03 (0.86-1.22)
Mean number sleeping in house (95% CI)	1.02 (0.94-1.09)	0.98 (0.92-1.03)	1.03 (0.92-1.13)
Mean asset index (95% CI)	0.93 (0.90-0.97)**	1.00 (0.96-1.04)	0.99 (0.93-1.07)

BAZ, body mass index-for-age-z scores; HAZ, height-for-age z scores; SD, standard deviation
**Odds ratio significant, p<0.01; *Odds ration significant, p<0.05
N-values reflect the total number of children, n represents the number of children in the risk group, estimates are adjusted using relevant weighting
